# A functional exercise program improves pain and health related quality of life in patients with fibromyalgia: a randomized controlled trial

**DOI:** 10.1186/s42358-024-00422-7

**Published:** 2024-10-24

**Authors:** Giovana Fernandes, Michele Nery, Sandra Mara Meireles, Rebeka Santos, Jamil Natour, Fabio Jennings

**Affiliations:** Rheumatology Division, Escola Paulista de Medicina, Universidade Federal de São Paulo, Rua Botucatu, 740, São Paulo, SP CEP: 04023-900 Brazil

**Keywords:** Fibromyalgia, Rehabilitation, Exercise, Functional training

## Abstract

**Background/objective:**

Fibromyalgia is a non-inflammatory syndrome characterized by generalized muscle pain, with other symptoms. Numerous forms of physical training for this population have been studied through high-quality randomized clinical trials involving strength, flexibility, aerobic conditioning and multicomponent exercise interventions. This research evaluated the effectiveness of a functional exercise program at reducing pain, improving functional capacity, increasing muscle strength as well as improving flexibility, balance and quality of life in individuals with fibromyalgia.

**Methods:**

Eighty-two women with fibromyalgia were randomized into two groups. The functional exercise group performed functional exercises in 45-minute sessions twice per week for 14 weeks. The stretching exercise group performed flexibility exercises with the same duration and frequency. Outcome measures were: visual analog scale for widespread pain; Fibromyalgia Impact Questionnaire for health-related quality of life; Timed Up and Go test for functional performance; one-repetition maximum for muscle strength, Sit and Reach test on Wells bench for flexibility; Berg Balance Scale for balance; SF-36 for general quality of life.

**Results:**

After the intervention, the functional exercise group had a statistically significant reduction in pain (interaction *p* = 0.002), and improvement in health-related quality of life measured by the Fibromyalgia Impact Questionnaire (interaction *p* < 0.001) and in general health state domain of SF-36 (interaction *p* = 0.043) compared to the stretching exercise group. No significant differences between groups were found regarding improvements in functional capacity, muscle strength, flexibility or balance.

**Conclusion:**

Functional exercise training was effective at reducing pain and improving quality of life in patients with fibromyalgia compared to stretching exercises.

**Trial registration:**

ClinicalTrials.gov Identifier: NCT03682588 First prospectively registered in March 2018.

## Background

Fibromyalgia (FM) is a non-inflammatory syndrome characterized by generalized muscle pain, with other symptoms beyond the musculoskeletal system [1]. Fibromyalgia is considered a pain-regulation disorder and affected individuals generally exhibit increased sensitivity to pain stimuli (hyperalgesia) and a low pain threshold (allodynia) [2]. Other frequent findings in FM include fatigue, sleep disorders, depression, anxiety, cognitive dysfunction, physical deconditioning and poor quality of life [3–5].

A large number of interventions have been developed for the treatment of FM, including medications, physiotherapy, exercises and educational programs [6–8]. Numerous forms of physical training for this population have been studied through high-quality randomized clinical trials involving strength, flexibility, aerobic conditioning and multicomponent exercise interventions [9–12].

Few clinical trials have been conducted involving functional exercise programs as an intervention for the treatment of chronic pain [13–16]. According to the American College of Sports Medicine (2009), functional exercise is work against resistance, with the force generated exerting direct benefits for both the performance of activities of daily living and movements related to the practice of sports. The relevance of functional exercise is in the broad application possibilities and the transference of the effects to daily activities [17–19].

The aim of functional training is to optimize the skills of an individual regarding the performance of a particular task [20]. Functional exercise protocols have been used to enhance athletic performance and diminish the incidence of injuries [21, 22], improve functioning in older people regarding the performance of activities of daily living [20, 23] and contribute to the rehabilitation of patients with chronic musculoskeletal diseases [24]. However, the literature offers no studies involving functional exercises for individuals with FM.

Therefore, the aim of the present study was to evaluate the effectiveness of a functional exercise program at reducing pain, improving functional capacity, increasing muscle strength as well as improving flexibility, balance and quality of life in individuals with FM.

## Methods

### Population

Eighty-two patients with FM according to the classification criteria of the American College of Rheumatology [25] were included in the study. The participants were women between 18 and 65 years of age with pain intensity between 4 and 8 on the 0-to-10-cm visual analog scale and on stable medication for FM for at least three months. The exclusion criteria were uncontrolled cardiorespiratory disease (coronary insufficiency, systemic arterial hypertension, pulmonary emphysema) or other condition that contraindicated the practice of physical exercise, severe psychiatric condition, uncontrolled diabetes mellitus, regular practice of physical exercise twice or more times per week in the previous three months and other inflammatory rheumatic disease associated.

This study received approval from the Institutional Ethics Committee. Patient recruitment was performed through a newspaper ad and at the rheumatology outpatient clinic of the Institution.

The patients were randomly assigned using a computer program to 2 groups: functional exercise group (FEG) and stretching exercise group (SEG). Opaque envelopes were used to ensure allocation concealment.

### Interventions

**Fig. 1:**
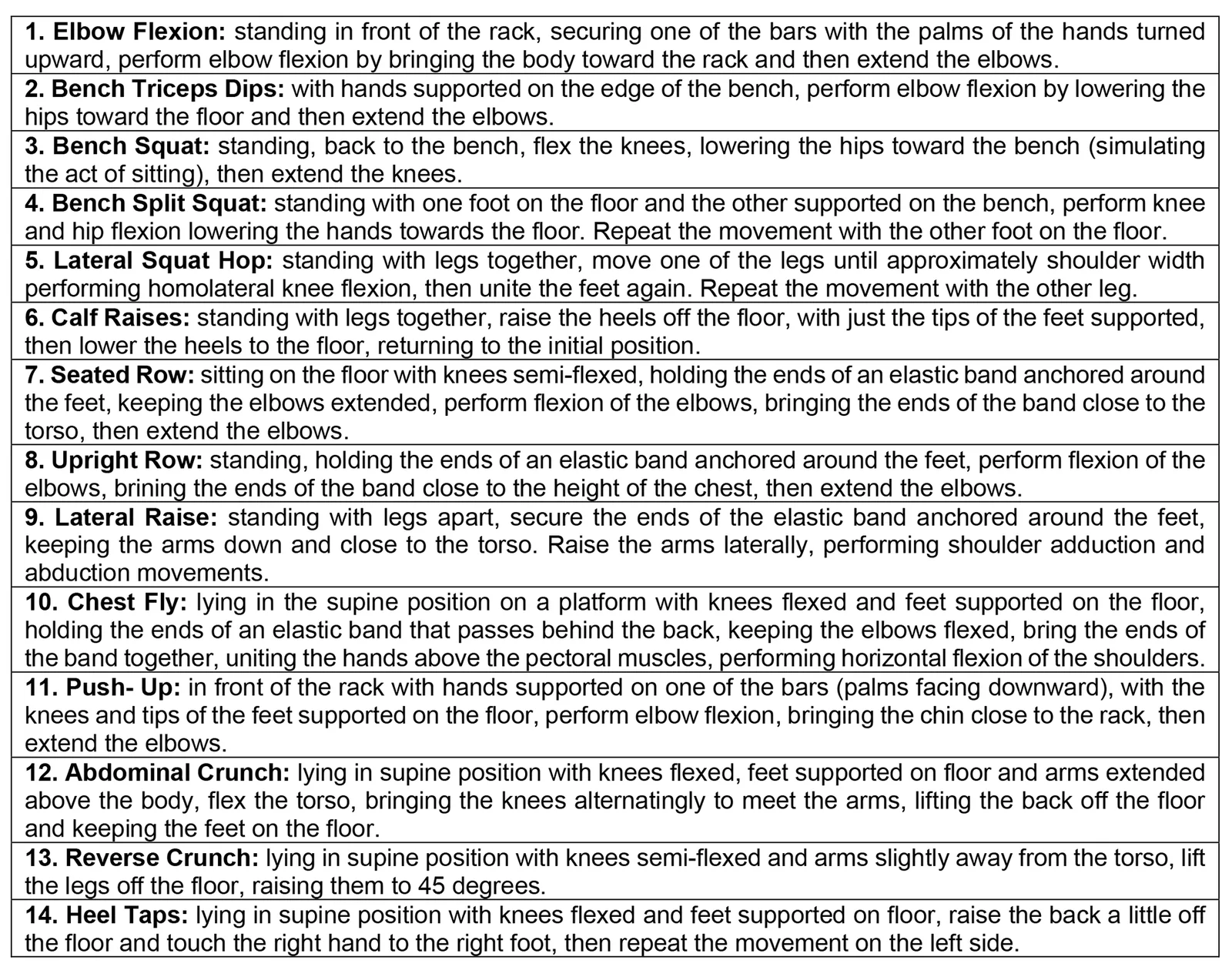
Functional exercise training program

The FEG was submitted to a functional exercise program in 45-minute sessions held twice per week for 14 weeks. The program consisted of 14 exercises, each of which was executed in two sets of ten repetitions, with a 30-second rest interval between sets (Fig. 1). Elastic band (Domyos^®^, Taiwan/China), measuring 170 cm in length and 15 cm in width were used during the execution of some exercises. Three colors corresponding to different resistance levels were used: yellow (light), grey (medium) and green (strong).

The resistance level (resistance band color) depended on the participant’s perceived exertion during a 10-repetition exercise set. When patients’ perceived exertion was rated as “somewhat hard” (still reasonably comfortable), resistance exercise intensity was increased using the next color band. The intensity of the exercises was reassessed every four weeks and the band was replaced with another of greater resistance depending on the progress of each patient [26].

**Fig. 2:**
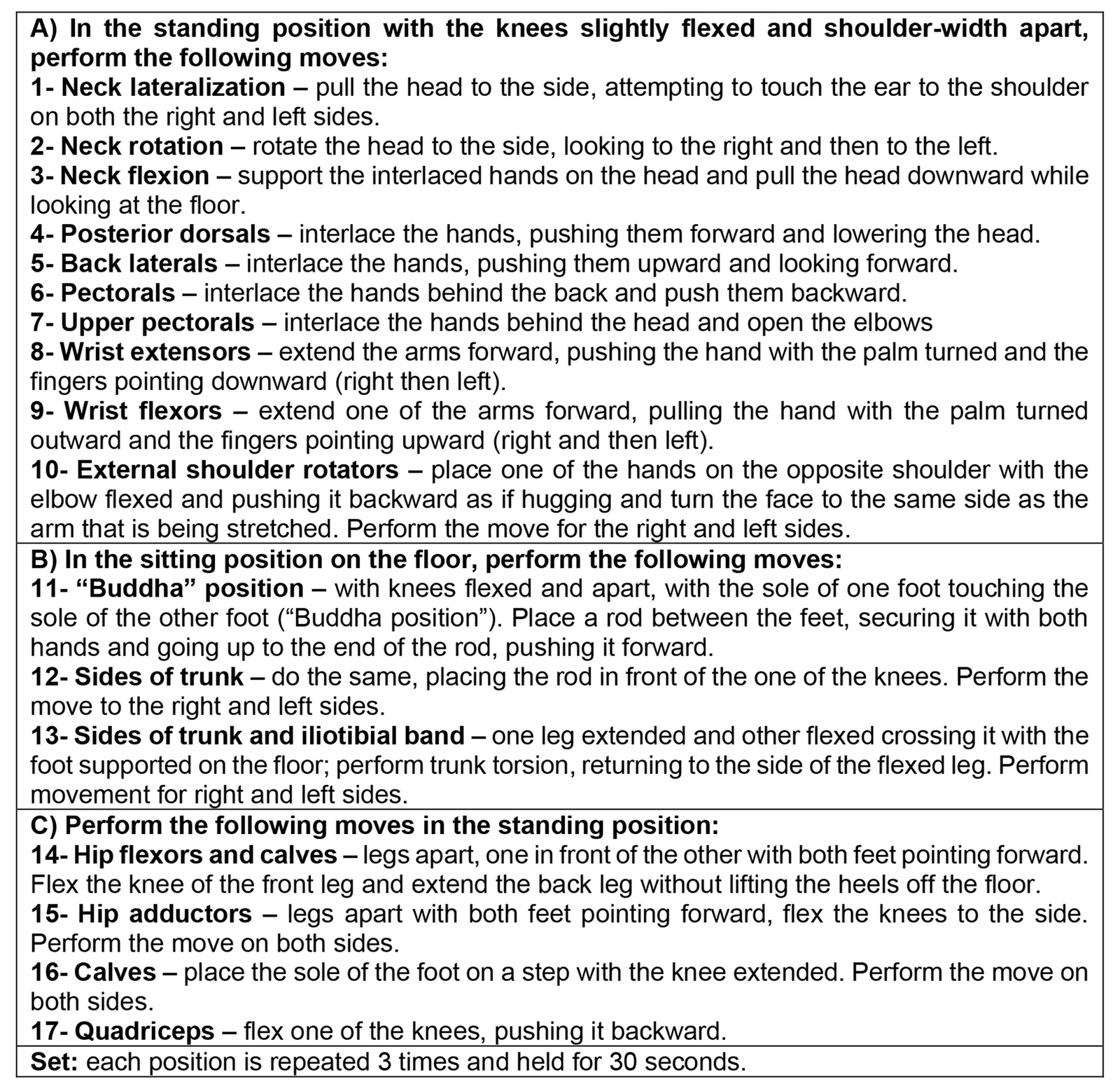
Stretching exercise training program

The SEG group was submitted to a stretching protocol used in a previous study involving an exercise program for individuals with FM [27]. The program consisted of 45-minute sessions held twice per week for 14 weeks and included 17 exercises, with each position held for 30 s. The exercises were chosen to promote global flexibility without increasing the heart rate. The following muscles were stretched: sternocleidomastoid, deltoids, wrist flexors and extensors, pectoral, triceps brachii, abdomen, biceps brachii, quadriceps, hamstrings, glutes, soleus and gastrocnemius (Fig. 2).

### Evaluations

The participants were evaluated prior to training (T0), after seven weeks (T7) and after 14 weeks (T14) of training, with follow-up evaluations after 26 weeks (T26); and 38 weeks (T38). The tests were performed in an environment with only the participant and evaluator. The evaluator was a physiotherapist who had undergone training for the administration of the tests and was blinded to the allocation of the participants.

The following data were collected during the initial medical evaluation: identification, age, weight, height, ethnicity, level of schooling, time since diagnosis, associated diseases, past history of physical exercise and medications. The following assessment tools were used for the other measures:

Visual analog scale (VAS) [28] for diffuse pain, which constituted a 10-cm horizontal line with 0 (absence of pain) printed at one end and 10 (unbearable pain) printed at the other end; Fibromyalgia Impact Questionnaire(FIQ) [29], which is composed of 10 items addressing quality of life related to the disease; Medical Outcome Survey Short Form 36 (SF-36) [30] to assess general quality of life; Timed Up and Go (TUG) Test [31] to assess functional performance; One repetition-maximum (1RM) [32] to assess muscle strength; Sit and Reach test [33] with the use of the Wells bench to assess flexibility; Berg Balance Scale [34, 35] to assess balance. The number of analgesics was recorded during the intervention period.

### Statistical analysis

The sample size was calculated using the VAS for pain as the main parameter [36]. Considering a standard deviation of 2.0 cm [37] (based on previous studies), a 90% power, 5% significance level and detectable difference of 2.0 cm on the VAS a minimum of 34 participants was determined for each group (total: 68). 20% was then added to compensate for possible dropouts, leading to a total of 82 participants (41 in each group).

Data analysis was performed with the aid of the SPSS software, version 15.0. Descriptive statistics (mean, standard deviation and 95% confidence interval) were performed for the characterization of the participants. Baseline continuous variables were compared between groups using the Student’s t-test for variables with normal distribution and the Mann-Whitney test for those with non-normal distribution.

Categorical variables were compared using the chi-square test. Repeated-measures analysis of variance (ANOVA) was used to evaluate the inter-group and intra-group response to treatment over time. Intention-to-treat analysis was used in the evaluation of the response to the intervention.

## Results

**Fig. 3:**
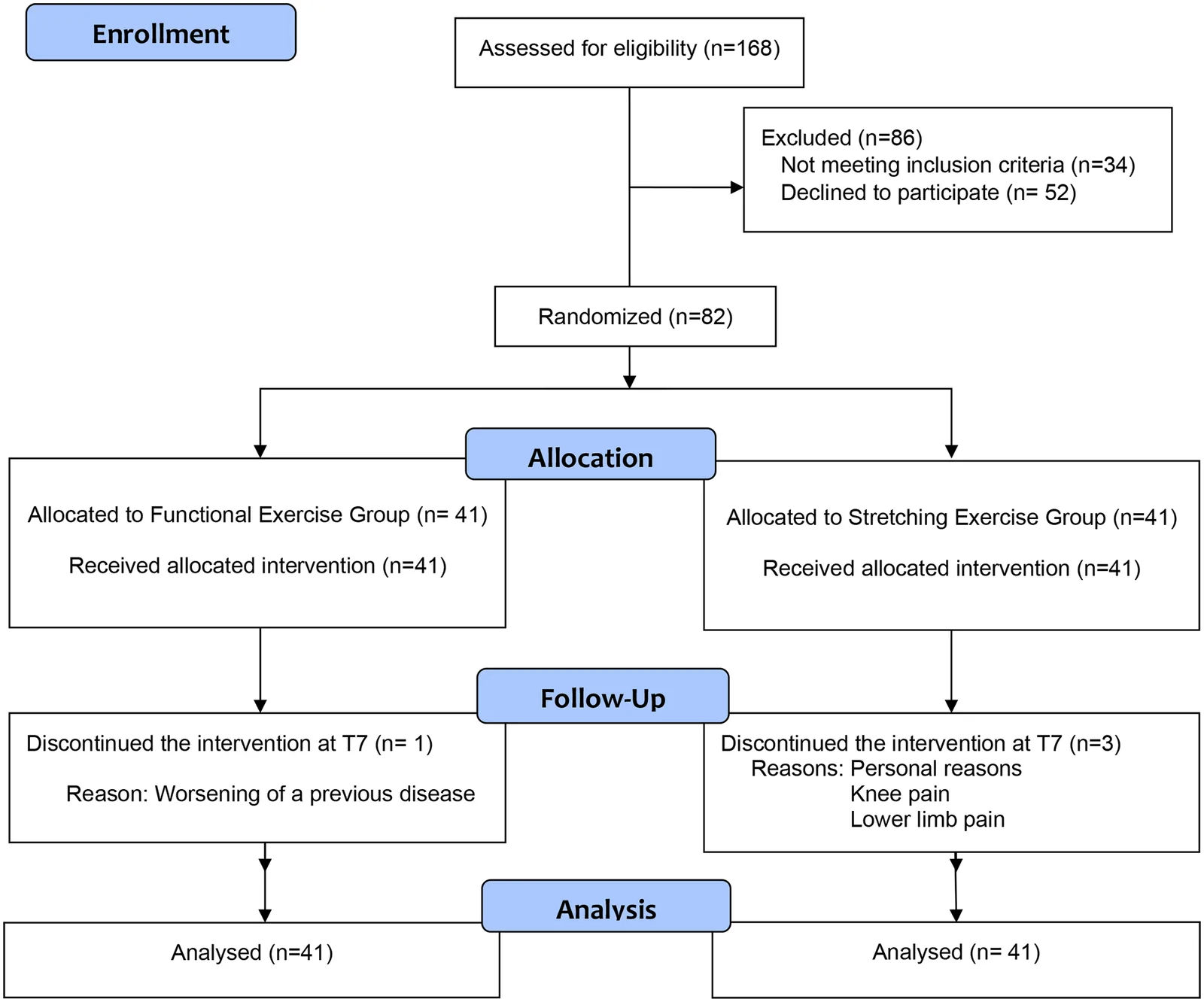
CONSORT flow diagram

A total of 168 patients were contacted and interviewed individually until 82 women were included in the study and randomized into the two groups: 41 in the FEG and 41 in the SEG. In the FEG, one participant discontinued the program because of a worsening of a previous disease. In the SEG, three participants discontinued the program because of personal reasons (1), knee pain (2) and lower limb pain (3). All dropouts were unrelated to the exercise programs. Nonetheless, intention-to-treat analysis was performed and all data were analyzed (Fig. 3).

The groups were similar regarding the clinical characteristics at baseline. The mean age was 47.8 years in the FEG and 55 years in the SEG. Mean schooling was 10.3 years in the SEG and de 12.2 years in the FEG. In both groups, 49% of the participants were white. The body mass index (BMI) was higher than 28 kg/m^2^ in both groups (Table 1).

**Table 1:** Baseline clinical and demographic characteristics of the patients with FM included in the study

	FEG (*n* = 41) Mean (SD)	SEG (*n* = 41) Mean (SD)
**Age (years)**	47.8 (11.3)	55.0 (8.0)
**Mean schooling (years)**	12.2 (3.7)	10.3 (4.9)
**Ethnicity**		
**White**	20 (49%)	20 (49%)
**Non-white**	21 (51%)	21 (51%)
**Body mass index (Kg/m** ^**2**^ **)**	29.1 (4.8)	28.5 (5.9)

*FEG* functional exercise group, *SEG* stretching exercise group, *SD* standard deviation

^#^*Mann-Whitney* test, **Student’s t-test, *Fisher’s exact test

There were statistically significant differences in pain between groups over time (interaction *p* = 0.002). The FEG had a statistically significant improvement in pain compared to the SEG at T7, T14 and T26. In the intra-group analyses, no statistically significant differences were found in pain in the SEG over time (intra-group *p* = 0.814), whereas a statistically significant difference was found in the FEG over time (intra-group *p* = 0.007).

There were statistically significant differences in health-related quality of life measured using the FIQ between groups over time (interaction *p* < 0.001). A statistically significant improvement in health status (in FIQ) was found in the FEG compared to the SEG, with differences between T7 and T14. In the intra-group analysis, no statistically significant differences were found in the FIQ scores in the SEG over time (intra-group *p* = 0.423), whereas significant differences were found in the FEG over time (intra-group *p* < 0.001) (Table 2).

**Table 2:** Pain and health-related quality of life scores in patients with fibromyalgia in the follow-up

Outcomes	Evaluation times Mean (SD)					*P* interaction	*P* intra-group
	T0	T7	T14	T26	T38		
**VAS pain (cm)**						0.002*	
**FEG**	7.0 (1.4)	4.9 (2.3)	4.8 (2.5)	5.0 (2.3)	5.8 (2.6)		0.007*
**SEG**	7.1 (1.3)	6.7 (1.9)	6.1 (2.4)	6.2 (2.4)	6.3 (2.3)		0.814
***P*** **between groups**	0.904	< 0.001*	0.010*	0.037*	0.642		
**FIQ**						< 0.001*	
**FEG**	6.55 (1.30)	5.03 (1.67)	4.39 (2.00)	5.26 (1.95)	5.32 (1.77)		< 0.001*
**SEG**	6.45 (1.47)	5.90 (1.80)	5.73 (1.77)	5.38 (1.92)	5.73 (1.96)		0.423
***P*** **between groups**	0.988	0.003*	< 0.001*	0.254	0.204		

*VAS* visual analogic scale, *FIQ* Fibromyalgia Impact Questionnaire, *SD* standard deviation, *FEG* functional exercise group, *SEG* stretching exercise group

*Analysis of variance, T0 = prior to training, T7 = after seven weeks, T14 = and after 14 weeks, T26 = after 26 weeks (follow up) and, T38 = after 38 weeks (follow-up)

No statistically significant differences between groups were found regarding the TUG test, flexibility test on the Wells bench or Berg Balance Scale. However, both groups exhibited statistically significant improvements over time (Table 3).

No statistically significant differences between groups were found regarding lower limb muscle strength (1RM) (interaction *p* = 0.522). However, both groups exhibited statistically significant improvements over time (intra-group *p* = 0.034). Likewise, no statistically significant differences between groups were found regarding upper limb muscle strength (1RM) (interaction *p* = 0.520), but both groups exhibited statistically significant improvements over time (intra-group *p* = 0.001) (Table 3).

Regarding the SF-36, no statistically significant differences in physical functioning, role-physical, role-emotional, social functioning, vitality or mental health domains were found between the two groups. However, statistically significant improvements were found on these domains in both groups over time (intra-group).

**Table 3:** Functional capacity, stretching, balance and muscle strength scores in patients with fibromyalgia

Outcomes	Evaluation time Mean (SD)					*P* interaction	*P* intra-group
	T0	T7	T14	T26	T38		
**TUG**						0.089	
**FEG**	7.91 (1.13)	7.35 (1.0)	7.26 (1.08)	7.27 (1.15)	7.53 (1.14)		< 0.001*
**SEG**	8.73 (1.83)	7.89 (1.4)	8.02 (1.72)	7.81 (1.55)	7.78 (1.45)		< 0.001*
***P*** **between groups**	0.237	0.237	0.237	0.237	0.237		
**Welss**						0.646	
**FEG**	21.94 (8.39)	25.24 (8.98)	26.21 (9.08)	25.16 (9.51)	24.09 (8.85)		< 0.001*
**SEG**	23.16 (1.47)	25.55 (9.03)	25.15 (8.99)	25.28 (9.52)	24.32 (8.69)		< 0.001*
***P*** **between groups**	0.738	0.738	0.738	0.738	0.738		
**Berg**						0.158	
**FEG**	54.0 (2.0)	55.0 (0.8)	55.3 (0.9)	54.8 (2.1)	55.1(1.4)		< 0.001*
**SEG**	51.9 (3.4)	53.7 (2.3)	54.2 (2.1)	53.08 (2.1)	54.1 (2.2)		< 0.001*
***P*** **between groups**	0.010*	0.010*	0.010*	0.010*	0.010*		
**1RM- lower limb**						0.522	
**FEG**	6.15 (1.33)	6.71 (1.33)	7.00 (1.47)	6.73 (1.60)	6.80 (1.69)		0.034*
**SEG**	5.78 (1.60)	6.00 (1.28)	5.95 (1.38)	6.07 (1.27)	6.05 (1.40)		0.034*
***P*** **between groups**	0.051	0.051	0.051	0.051	0.051		
**1RM- upper limb**						0.520	
**FEG**	12.4 (5.9)	14.9 (5.4)	15.5 (6.6)	14.2 (5.9)	13.5 (5.9)		< 0.001*
**SEG**	9.8 (5.1)	13.0 (6.6)	12.4 (7.0)	12.4 (6.6)	10.5 (5.3)		< 0.001*
***P*** **between groups**	0.004*	0.004*	0.004*	0.004*	0.004*		

*TUG* Time Up and Go test, *RM* One repetition-maximum; *SD* standard deviation, *FEG* functional exercise group, *SEG* stretching exercise group

*Analysis of variance; T0 = prior to training, T7 = after seven weeks, T14 = and after 14 weeks, T26 = after 26 weeks (follow up) and, T38 = after 38 weeks (follow-up)

Likewise, no statistically significant differences between groups were found for the bodily pain domain, but statistically significant improvements in bodily pain were found in both groups over time (intra-group *p* = 0.003). For the general health state domain, statistically significant differences were found between the two groups over time with greater improvement in the FEG (interaction *p* = 0.043). In the intra-group analyses, statistically significant differences were found in the FEG (intra-group *p* = 0.046), whereas no statistically significant differences were found over time in the FEG (intra-group *p* = 0.654). For the same domain, in the intergroup analysis, improvement was greater in the FEG compared to the SEG at T7, T14 and T38 (Table 4).

The attendance to the programs was similar in the two groups (78.6%). Regarding the number of analgesics taken during the follow-up period, no statistically significant differences between groups were found over time.

**Table 4:** SF-36 scores (general quality of life) in groups at different evaluation times

SF-36 domains	Evaluation time Mean (SD)					*P* interaction	*P* intra-group
	T0	T7	T14	T26	T38		
**Physical functioning**						0.443	
**FEG**	46.7 (22.5)	56.6 (20.9)	63.2 (21.1)	63.7 (23.0)	61.8 (22.5)		< 0.001*
**SEG**	43.8 (125.9)	51.0 (24.1)	55.6 (26.3)	54.3 (23.0)	53.0 (23.8)		< 0.001*
***P*** **between groups**	0.054	0.054	0.054	0.054	0.054		
**Role-physical**						0.056	
**FEG**	12.8 (28.6)	32.09 (42)	35.4 (44)	37.2 (42.6)	39.3 (42.7)		< 0.001*
**SEG**	16.5 (29.9)	26.2 (36)	43.3 (46.1)	26.8 (39.3)	21.5 (34)		< 0.001*
***P*** **between groups**	0.055*	0.055*	0.055*	0.055*	0.055*		
**Bodily pain**						0.312	
**FEG**	47.9 (30.8)	56.2 (22)	62.6 (25.4)	58.0 (26.4)	56 (27.3)		0.003*
**SEG**	39.8 (27.5)	45.4 (22.7)	47.9 (22.6)	43.0 (26.0)	42.9 (23.4)		0.003*
***P*** **between groups**	0.002*	0.002*	0.002*	0.002*	0.002*		
**General health state**						0.043*	
**FEG**	48.9 (24.7)	52.6 (23.5)	57.9 (22)	51.5 (27.8)	56.0 (25.3)		0.046*
**SEG**	43.2 (18.5)	45.6 (19.7)	46.5 (22.4)	49.1 (22.2)	44.5 (22.4)		0.654
***P*** **between groups**	0.061	0.047	0.004*	0.313	0.006*		
**Vitality**						0.648	
**FEG**	36.5 (22.5)	47.2 (20.6)	50.9 (21.7)	50.1 (24.9)	47.5 (23)		< 0.001*
**SEG**	32.3 (22.5)	41.2 (21.4)	42.1 (22.5)	39.4 (22.3)	41.2 (22.3)		< 0.001*
***P*** **between groups**	0.002*	0.002*	0.002*	0.002*	0.002*		
**Social functioning**						0.066	
**FEG**	55.7 (24.5)	64.5 (25.4)	63.3 (27.9)	66.5 (26.4)	57.8 (31.1)		< 0.015*
**SEG**	48.3 (28.1)	59.9 (23.8)	57.4 (29.1)	56.0 (29.7)	63.7 (29.8)		< 0.015*
***P*** **between groups**	0.152	0.152	0.152	0.152	0.152		
**Role-emotional**						0.314	
**FEG**	31.7 (42.2)	39.8 (46.7)	47.1 (46.0)	36.5 (44)	43 (43.7)		< 0.017*
**SEG**	25.8 (40.5)	39.0 (46.7)	46.9 (42.3)	47.8 (44.7)	32.5 (40.5)		< 0.017*
***P*** **between groups**	0.281	0.281	0.281	0.281	0.281		
**Mental health**						0.075	
**FEG**	48.4 (20.9)	57.2 (15.8)	58.9 (21.2)	56.2 (23.1)	55.3 (20.9)		< 0.017*
**SEG**	41.4 (20.2)	51.1 (18.2)	51.2 (17.7)	56.9 (20.4)	54.0 (24.5)		< 0.017*
***P*** **between groups**	0.052	0.052	0.052	0.052	0.052		

*SF-36* The 36-Item Short Form Health Survey questionnaire, *SD* standard deviation, *FEG* functional exercise group, *SEG* stretching exercise group

*Analysis of variance; T0 = prior to training, T7 = after seven weeks, T14 = and after 14 weeks, T26 = after 26 weeks (follow up) and, T38 = after 38 weeks (follow-up)

## Discussion

The present study found positive results from a functional exercise program on pain reduction and improvement in health-related quality of life in subjects with FM. An improvement in the general health state domain of overall quality of life was also found in comparison to the subjects who participated in the stretching exercise program.

The benefits obtained in this study involving physical exercise interventions for the population with FM are compatible with the scientific literature. There is strong evidence of the importance of an active lifestyle in managing the symptoms of FM and assisting in the maintenance of functional capacity [38]. Indeed, exercise is considered the main non-pharmacological strategy in the treatment of FM [39].

Despite the difference in mean age, the two groups had similar scores for the other variables of interest. While the difference in age was statistically significant, it did not exert an influence on the results, as the comparisons of the variables measured over time were performed using statistical tests corrected for age and all statistically significant differences remained significant after the Bonferroni correction.

Studies involving exercise protocols as interventions report that regular aerobic exercise leads to improvements in pain, mood and physical functioning in individuals with FM [40, 41]. Strength training interventions have also had a positive impact, diminishing pain and depression, improving muscle strength and enhancing one’s performance on activities of daily living [42].

In the present study, the participants in the intervention group, who were submitted to training with functional exercises, reported a statistically significant reduction in pain measured using the VAS as well as a significant improvement in health-related quality of life measured using the FIQ.

Improvements in pain were found seven and 14 weeks after the onset of the intervention and remained stable for 12 weeks after the end of training. At 24 weeks after the end of training, however, no significant benefits remained, demonstrating that such benefits would only persist if the individuals continued performing the physical activity.

Improvements in health-related quality of life were found seven and 14 weeks after the onset of the intervention, whereas no statistically significant differences were found 12 and 24 weeks after the end of training. The participants in the group submitted to stretching exercises reported no improvements in pain or health-related quality of life over time, which demonstrates the effectiveness of functional exercises with regards to these variables.

Jones et al. [42] and Larsson et al. [43] conducted studies with a similar design to that of the present investigation, comparing a progressive resistance exercise program to relaxation and stretching exercises in patients with FM, and found similar results, with a significant decrease in VAS pain and FIQ scores only in the group submitted to strengthening exercises.

Regarding functional capacity assessed using the TUG test, both groups in the present study presented statistically significant improvement over time, even at the follow-up evaluations 12 and 24 weeks after the end of training. Two previous studies involving strengthening exercises for individuals with FM used other tests to measure aspects related to functional capacity. Larsson et al. [43] used the Six-Minute Walk Test (TC6) and Kingsley et al. [44] used the Continuous Scale – Physical Function Performance test. In both studies, functional performance was significantly better in the groups submitted to strengthening exercises; however, the control groups were not submitted to physical exercise interventions. In the present study, both groups exhibited improvements on the TUG test, which may have been related to the good functional performance of both groups since the onset of treatment as well as the adaptation of the participants to the test throughout the course of the evaluations, as the test was repeated three times at each evaluation and the best result was considered in the analysis.

Both groups also exhibited statistically significant improvements over time with regard to flexibility, which was assessed using the Sit and Reach test on the Wells bench. Few studies involving the population with FM have used tests to measure flexibility. Jones et al. [42] evaluated the effectiveness of a strengthening exercise program compared to a flexibility exercise program and found similar results to those of the present investigation, with no statistically significant difference between groups and improvement in both groups over time.

Both groups exhibited statistically significant improvements in balance measured by the Berg Balance Scale over time. In a study conducted to identify the effectiveness of balance exercises for improvements in functioning and quality of life among individuals with FM, Kibar et al. [45] compared the effects of a mixed exercise program that included flexibility and balance training compared to flexibility training alone. Balance was measured using both the Berg Balance Scale and a kinesthetic ability training device. The authors only found improvement in the group submitted to flexibility and balance exercises, which differs from the present findings.

Fibromyalgia is associated with balance problems and an increase in the frequency of falls. However, further studies are needed to identify the relative contributions of neural and musculoskeletal impairments on postural stability in this population [46, 47]. Moreover, the effects of diverse exercise modalities on postural stability are not yet clear [45].

Regarding upper and lower limb strength measured using the 1RM test, increases were found in both groups over time in the present study. The gain in strength in the stretching exercise group may be due to the adaptation to the 1RM test throughout the course of the evaluations as well as the fact that the functional program was not composed of progressive resistance exercises.

Positive results were found regarding general quality of life in both groups over time. Sanudo et al. [39] found positive results for some SF36 domains in individuals with FM submitted to a mixed training intervention that included aerobic exercises combined with strengthening and flexibility exercises. The results differed somewhat from those of the present investigation, as significant improvements in the study by Sanudo et al. [39] only occurred in the physical functioning, general health state, vitality and mental health domains.

Sevimli et al. [48] compared the effects of three exercise programs on individuals with FM. The first group performed strengthening and flexibility exercises at home, the second group performed aerobic exercises and the third group performed aerobic exercises in an aquatic environment. The authors found similar results to those of the present investigation regarding SF36 scores, as all groups exhibited improvements over time, with no statistically significant inter-group differences.

Individuals with FM find it difficult to adhere to a physical exercise program [49]. It is therefore difficult to get them to attend 100% of the scheduled training sessions.

The patients included in the study exhibited good acceptance of the adapted functional exercise training proposal (95.2% adherence to treatment). The set involved seven free exercises with body weight used as the load, three exercises involving wall-bar support also with body weight as the load, and four exercises involving elastic straps with resistance altered progressively based on individual progress and tolerance. We believe that the positive acceptance and tolerance on the part of the patients was due to the following: the non-use of dumbbells, weights and body-building machines, which may give the impression that the exercises are lighter and easy to perform; all patients were on stable FM medication for at least three months; the patients had pain intensity between 4 and 8 on the VAS that diminished throughout training (Table 2); the patients were accompanied by a specialized team (physician and exercise instructor specialized in rheumatology); the patients performed exercise with load progression based on the progress and tolerance of each patient; and the exercises were performed in small groups with the complete attention of the instructor. In the literature, we found that the effects of functional exercise programs have been observed in athletes [21, 22], the older popluation [20, 23], and in the field of rehabilitation, such as in patients with chronic low back pain [24], knee osteoarthritis [50] and psoriatic arthritis [51].

One limitation of the present study regards the choice of the exercise protocol, as functional training is a broad concept and only movements that simulated activities of daily living were chosen, limiting the effects of the program to individuals who perform these specific physical activities. Another limitation regards the progression of load during the functional exercises. The decision regarding load was based on the participants’ perception of effort in relation to the resistance of the elastic band, with no objective load value considered for the progression.

This study showed that the functional exercise training program used was capable of diminishing pain and improving quality of life related to the disease in individuals with FM after seven weeks and these benefits were maintained for up to 12 weeks after the end of the intervention. However, the benefits did not persist for up to 24 weeks after the intervention, which underscores the need to continue the exercises. The reduction in pain through functional exercise training is the most important finding of this study, as pain is the major symptom in FM.

## Conclusion

The functional exercise training program was effective at reducing pain and improving the quality of life in patients with fibromyalgia when compared to stretching exercises.

## Data Availability

The datasets used and/or analyzed during the current study are available from the corresponding author on reasonable request.

## Abbreviations

ANOVA: Repeated-measures analysis of variance
BMI: body mass index
FM: fibromyalgia
FEG: functional exercise group
FIQ: Fibromyalgia Impact Questionnaire
SF-36: Medical Outcome Survey Short Form 36
SEG: stretching exercise group
SPSS: Statistical Package for Social Science for Windows
VAS: Visual Analog Scale
TUG: Timed Up and Go
1RM: One repetition-maximum
